# Integrative analysis of immune‐related multi‐omics profiles identifies distinct prognosis and tumor microenvironment patterns in osteosarcoma

**DOI:** 10.1002/1878-0261.13160

**Published:** 2022-01-01

**Authors:** Deyao Shi, Shidai Mu, Feifei Pu, Jianxiang Liu, Binlong Zhong, Binwu Hu, Na Ni, Hao Wang, Hue H. Luu, Rex C. Haydon, Le Shen, Zhicai Zhang, Tong‐Chuan He, Zengwu Shao

**Affiliations:** ^1^ Department of Orthopaedics Union Hospital Tongji Medical College Huazhong University of Science and Technology Wuhan China; ^2^ Molecular Oncology Laboratory Department of Orthopaedic Surgery and Rehabilitation Medicine The University of Chicago Medical Center IL USA; ^3^ Institution of Hematology Union Hospital Tongji Medical College Huazhong University of Science and Technology Wuhan China; ^4^ Ministry of Education Key Laboratory of Diagnostic Medicine Department of Clinical Biochemistry the School of Laboratory Medicine Chongqing Medical University China; ^5^ Department of Surgery The University of Chicago Medical Center IL USA

**Keywords:** DNA methylation, osteosarcoma, prognostic risk model, transcriptomics, tumor immunology, tumor microenvironment

## Abstract

Osteosarcoma (OS) is the most common primary malignancy of bone. Epigenetic regulation plays a pivotal role in cancer development in various aspects, including immune response. In this study, we studied the potential association of alterations in the DNA methylation and transcription of immune‐related genes with changes in the tumor microenvironment (TME) and tumor prognosis of OS. We obtained multi‐omics data for OS patients from the Therapeutically Applicable Research to Generate Effective Treatments (TARGET) and Gene Expression Omnibus (GEO) databases. By referring to curated immune signatures and using a consensus clustering method, we categorized patients based on immune‐related DNA methylation patterns (IMPs), and evaluated prognosis and TME characteristics of the resulting patient subgroups. Subsequently, we used a machine‐learning approach to construct an IMP‐associated prognostic risk model incorporating the expression of a six‐gene signature (*MYC*, *COL13A1*, *UHRF2*, *MT1A*, *ACTB*, and *GBP1*), which was then validated in an independent patient cohort. Furthermore, we evaluated TME patterns, transcriptional variation in biological pathways, somatic copy number alteration, anticancer drug sensitivity, and potential responsiveness to immune checkpoint inhibitor therapy with regard to our IMP‐associated signature scoring model. By integrative IMP and transcriptomic analysis, we uncovered distinct prognosis and TME patterns in OS. Finally, we constructed a classifying model, which may aid in prognosis prediction and provide a potential rationale for targeted‐ and immune checkpoint inhibitor therapy in OS.

## Graphical abstract

Epigenetic regulation plays a pivotal role during cancer development, including immune response within the tumor microenvironment (TME). We studied the potential association of DNA methylation and the transcriptional alterations of immune‐related genes in the TME of osteosarcoma. Following integrative analysis of multi‐omics data, we developed a prognostic risk model that may provide a potential rationale for targeted therapy and immunotherapy for patients with osteosarcoma.

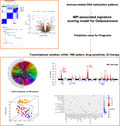

**Abbreviations**
AIC: Akaike's information criterion
AUC: area under the ROC curve
BMIQ: beta‐mixture quantile dilation
C‐index: concordance index
CTL: cytotoxic T lymphocytes
DCA: decision curve analysis
DEG: differentially expressed gene
DMP: differentially methylated probe
EMBL‐EBI: European Molecular Biology Laboratory's European Bioinformatics Institute
FDR: false discovery rate
GDSC: Genomics of Drug Sensitivity in Cancer
GEO: Gene Expression Omnibus
GO: gene ontology
GSEA: gene set enrichment analysis
GSVA: gene set variation analysis
IC50: half‐maximal inhibitory concentration
ICI: immune checkpoint inhibitor
ImmPort: The Immunology Database and Analysis Portal
IMP: immune‐related DNA methylation pattern
KEGG: Kyoto Encyclopedia of Genes and Genomes
LASSO: least absolute shrinkage and selection operator
MeTIL: methylation of tumor‐infiltrating lymphocytes
NES: normalized enrichment score
OS: osteosarcoma
PPI: protein‐protein interaction
RNA‐Seq: RNA sequencing
ROC: receiver operating characteristic
SCNA: somatic copy number alternation
TARGET: Therapeutically Applicable Research to Generate Effective Treatments
TCR: T‐cell receptor
TIDE: tumor immune dysfunction and exclusion
TIS: T‐cell inflamed signature
TMB: tumor mutation burden
TME: tumor microenvironment
TNB: tumor neoantigen burden
TPM: transcript per million
t‐SNE: t‐distributed stochastic neighbor embedding
TSS: transcription start site
UCSC: University of California, Santa Cruz
UTR: untranslated region
VST: variance stabilizing transformation

## 1 Introduction

Human osteosarcoma (OS) is one of the most common and aggressive primary bone tumors, which is most prevalent in adolescents and young adults, and primarily affects the long bones, such as the distal femur, proximal tibia, and humerus [1, 2]. Although definitive surgical resection combined with adjuvant chemotherapy has remarkably improved the prognosis of patients with localized OS, ~20% of OS patients suffer from pulmonary metastatic disease at initial diagnosis [3]. The 5‐year survival rate of OS patients with chemotherapy resistance or metastasis is ~20–30% [4, 5, 6]. To effectively improve OS patients' survival, it is imperative to identify novel biomarkers that can predict clinical outcomes and the treatment sensitivity of OS.

DNA methylation alterations play a crucial role in cancer development. During oncogenesis, the hypermethylation promoter region can lead to the epigenetic silencing of tumor suppressor genes, whereas aberrant DNA methylation of nonpromoter elements is an important contributor to intratumoral heterogeneity. Thanks to their highly conserved characteristics, some DNA methylation‐related biomarkers have been identified for the early diagnosis or prognosis prediction of cancer [7, 8, 9, 10].

The important role of the immune system in suppressing oncogenesis and progression has made immunotherapy the fourth pillar in cancer management, along with surgery, chemotherapy, and radiotherapy. Nevertheless, not all patients fully benefit from such therapies, and many of them, including OS patients, fail to achieve complete responses or suffer frequent relapses. Epigenomic signatures in immune and cancer cells are promising predictors of clinical outcomes with immunotherapy. Jeschke et al. [11] profiled DNA methylation markers to identify a methylation of the tumor‐infiltrating lymphocytes (MeTIL) signature, thus evaluated the local tumor immune response and improved the prognostic accuracy for patients with breast cancer and other cancers. Dejaegher et al. [12] evaluated the relationship of epigenetic glioblastomas subgroups with immune cell infiltrations and survival, and validated the importance of DNA methylation profiles in stratifying patients for immunotherapy trials.

In this study we clustered OS patients into three immune‐related DNA methylation patterns (IMPs) that were distinctly related to prognosis, and then analyzed the diverse clinicopathological characteristics and tumor immune microenvironmental landscape of patients in different IMPs. A six‐gene IMP‐associated signature scoring model and nomogram were constructed as a robust prognostic model with favorable predictive performance. After evaluating the genetic and epigenetic features of IMPs and the correlations between IMPs and somatic copy number alternations (SCNAs) in OS, we demonstrated that the IMP‐associated signature scoring model was capable of distinguishing tumor microenvironment (TME) subtypes, predicating clinical outcomes, and assessing therapeutic sensitivities in targeted therapy and immunotherapy of OS.

## 2 Materials and methods

### 2.1 Data selection and acquisition

The acquisition and use of patients' genetic data and clinical information strictly followed the TARGET Publication Guidelines (https://ocg.cancer.gov/programs/target/target‐publication‐guidelines). We obtained clinical information, RNA‐seq (gene expression), DNA methylation array, and DNA copy number variation data of OS patients from the Therapeutically Applicable Research to Generate Effective Treatments (TARGET, https://ocg.cancer.gov/programs/target). RNA‐seq data of count and TPM (transcript per million) formats were extracted from a total of 98 patients (96 of 98 with matched clinical information). The *Homo sapiens* GRCh38.103.chr.gtf annotation file from Ensembl was downloaded for gene symbol annotation corresponding to Ensembl ID [13]. The deseq2 r package was applied to filter out low‐abundance profiles and normalize RNA‐seq counts data [14]. Variance stabilizing transformation (VST) data processed by deseq2 was used for downstream analysis such as the unsupervised clustering of patients, gene set variation analysis (GSVA), and scoring model construction. Beta value matrices based on the Illumina (San Diego, CA) HumanMethylation450 BeadChip assay were extracted from a total of 86 patients (84 of 86 with matched clinical information). The champ r package was used to normalize (using BMIQ function) the DNA methylation profiles [15, 16]. We utilized the IlluminaHumanMethylation450kanno.ilmn12.hg19 r package for genomic annotation of CpG sites [17]. DNA copy number segmentation profiles were extracted from a total of 88 patients. Clinical information of OS patients in the TARGET cohort was collected, including patient ID, gender, race, age, relapse during the follow‐up, vital status, overall survival follow‐up time, relapse‐free survival follow‐up time, metastasis at diagnosis, and histologic response to chemotherapy.

We collected publicly available datasets for independent validation. The gene expression profile (processed microarray data) of OS patients in the GSE21257 dataset was downloaded from the GEO database (www.ncbi.nlm.nih.gov/geo) [18, 19]. DNA methylation profiles (raw IDAT data) of OS patients in the E‐MTAB‐9875 dataset and chondrosarcoma patients in the E‐MTAB‐7263 dataset were downloaded from the ArrayExpress of the EMBL‐EBI database (www.ebi.ac.uk) [20, 21, 22] and then processed through the champ r package. Clinical information of these cohorts was collected when available, including patient ID, age, gender, vital status, overall survival follow‐up time, metastasis status, metastasis‐free survival follow‐up time, histologic response to chemotherapy, and tumor grade. Detailed information for the TARGET OS and the three validation datasets are documented in Table S1. All patient cohorts used in this study were obtained from publicly available datasets that were collected with patients' informed consent. The study methodologies conformed to the standards set by the Declaration of Helsinki, and were approved by the local Ethics Committee.

We constructed the immunologic gene list (Table S1) by combining gene sets from two previously reported databases: the ImmuneSigDB (via MSigDB Collections) and ImmPort [23, 24].

### 2.2 Clustering immune‐related DNA methylation patterns of OS

To identify the immune‐related methylation patterns in OS, we applied an unsupervised consensus clustering algorithm. Before clustering, we first performed univariate Cox regression analysis on the methylation level (beta values) of all CpG sites in 84 patients with matched DNA methylation profiles and overall survival follow‐up data. CpG sites that were associated with patients' OS were retained (*P* < 0.05). Next, according to the genomic annotation of CpG sites, we selected CpG sites corresponding to genes that were subject to immunologic signature gene sets. Finally, we utilized the consensusclusterplus r package to carry out an unsupervised consensus clustering on these immune‐related CpG sites based on the *K*‐means algorithm, and the resampling was set to be 1000 repetitions to ensure clustering stability [25]. The distance matrix of consensus clustering was applied for a silhouette analysis to assess how similar an individual was matched to its assigned cluster compared to other clusters by using the cancersubtypes r package [26].

### 2.3 Identification of differentially expressed genes and differentially methylated CpG sites

The deseq2 r package was applied to process RNA‐seq counts data and then identify differentially expressed genes (DEG) between two groups. The differential expression threshold was defined with a fold‐change of threshold at 1.5 and an adjusted *P* value (false discovery rate, FDR) < 0.05. The champ r package was applied to normalize beta‐value matrices and then identify differentially methylated probes (DMP) between two groups. The differential methylation threshold was defined with a 15% differential (|Δβ| > 0.15) and an adjusted *P* value (FDR) < 0.05 [27]. enhancedvolcano and pheatmap r packages were utilized for the visualization of volcano plot and heatmap [28, 29].

### 2.4 Correlation between DNA methylation and gene expression

A total of 83 OS patients with matched methylation and expression data were used for correlation analysis. According to genomic annotation of CpG sites, we selected probes that were located in the promoter region including TSS1500, TSS200, 5′UTR, and 1st Exon. Both cis‐ and trans‐regulations were analyzed on the DEGs. Pearson correlation coefficients were calculated between the expression value and the methylation level of each CpG site. Correlation was with significance if the correlation coefficient was greater than 0.3 and the adjusted *P* value (FDR) was less than 0.05.

### 2.5 Evaluation of TME cell infiltration abundance

We used gene expression in TPM format (not log‐transformed) and applied three independent algorithms to evaluate the TME cell infiltration abundance: (a) the CIBERSORTx algorithm was conducted through its online tool (https://cibersortx.stanford.edu/) to quantify the abundance of 22 types of TME infiltrating cells. We set parameters as follows: 100 times for permutation test, batch correction – bulk mode, RNA‐seq data without quantile normalization, and output scores using the absolute mode [30]; (b) the quanTIseq algorithm was applied to quantify the abundance of 10 types of TME‐infiltrating cells via the immunedeconv r package [31, 32]; (c) the xCell algorithm was applied to evaluate the overall TME infiltration extent including immune and stromal infiltration levels via the immunedeconv r package [33].

### 2.6 Functional enrichment analysis

The clusterprofiler and goplot r packages were used for overrepresentation analysis, preranked gene set enrichment analysis (GSEA), and visualization [34, 35, 36]. Gene sets of hallmarks, Gene Ontology (GO) Biological Process section, Kyoto Encyclopedia of Genes and Genomes (KEGG) pathway, and Reactome pathway were analyzed in this study [37, 38, 39, 40]. A *P*‐value < 0.05, FDR < 0.05 and | normalized enrichment score (NES) | > 1.0 was considered as significant for the preranked GSEA. The nonparametric GSVA of patients among multiple groups was conducted by using the gsva r package [41].

### 2.7 Construction and validation of IMP‐associated signature scoring model

In order to evaluate the IMP and prognosis of individual patients with OS, we constructed an IMP‐associated signature scoring model by using TARGET datasets as the training cohort and validated it using an external dataset (GSE21257) as the testing cohort. We first evaluated the prognostic significance of DEGs between IMPs based on the univariate Cox regression analysis and selected genes associated with patients' overall survival (*P* < 0.05). Next, we applied Lasso (least absolute shrinkage and selection operator) regression analysis to narrow the range of candidates and screened a relatively small group of genes with a nonzero regression coefficient by using the glmnet r package [42]. A stepwise regression using the Akaike's information criterion (AIC) method was further performed to construct a signature scoring model with an optimal number of genes. For each individual, the risk score was calculated as the sum of Exp*_i_* × Beta*_i_*, where Exp means normalized gene expression, Beta means regression coefficient and *i* means the candidate gene in the scoring model. We conducted time‐dependent receiver operating characteristic (ROC) curves analysis and Kaplan–Meier survival analysis to evaluate the prognostic significance and accuracy of the IMP‐associated signature scoring model through the survivalroc and survminer r packages. Harrell's concordance index (*C*‐index) was calculated by using the survcomp r package. The optimal cutpoint for dividing patients into high‐ and low‐IMP_Risk group was defined as the value with the largest Youden index in the time‐dependent ROC curve of the median overall survival time. For each individual, the IMP_Risk score along with clinicopathological characteristics were analyzed by univariate and multivariate Cox regression analyses. Identified independent prognostic factors were then selected to construct a nomogram for the prediction of the likelihood of overall survival by using the rms r package [43]. Furthermore, we applied the rms and rmda r packages to produce calibration plots and performed decision curve analysis (DCA) to evaluate the nomogram [44].

### 2.8 Identification of recurrent regions with somatic copy number alteration

To determine significantly amplified or deleted regions of SCNA, we applied GISTIC 2.0 to analyze DNA copy number segmentation profiles [45]. The full analysis process of GISTIC 2.0 was conducted on the GenePattern platform [46]. We set parameters of GISTIC 2.0 as follows: a noise threshold of 0.3, a focal length cutoff of 0.5, a confidence level of 90%, a *q*‐value threshold of 0.25, a copy‐ratio cap of 1.5, and arm‐level peel‐off mode enabled. GISTIC 2.0 identified significant amplification or deletion and listed the “wide peak” region of SCNA. We applied the genomicranges r package to determine genes that overlapped in any “wide peak” region with a residual *q* value less than 0.05 (Residual *q*‐values: The *q*‐value of the peak region after removing segments shared with higher peaks) [47]. The results of GISTIC 2.0 analysis were visualized by the maftools r package [48].

### 2.9 Evaluation of protein–protein interaction

For the genes sets identified significantly associated with IMP risk scores, we constructed a protein–protein interaction (PPI) network based on the STRING database and applied the cytoscape plugins for evaluation [49, 50]. cytohubba was utilized to identify hub genes with top degree in the network [51]. mcode was utilized for identification of the most highly correlated subclusters [52].

### 2.10 Assessment of IMP‐associated signature scores on drug sensitivity

The database of the Genomics of Drug Sensitivity in Cancer (GDSC) provided a landscape of pharmacogenomic interactions in cancer [53]. GDSC collected the transcriptomic profiles for about 1000 cancer cell lines. Compounds were screened on these cancer cells and corresponding dose responses were measured as IC50 or area under the curve (AUC) quantitatively. We obtained normalized gene expression data, drug response quantification (AUC), and information on putative targets or pathways of drugs from the GDSC database. Spearman correlation analysis was performed to assess the correlation between drug sensitivity and the IMP risk score. A drug is considered sensitive to the IMP risk score when the *R*_S_ is less than −0.15 and the adjusted *P* value (FDR) is less than 0.05.

### 2.11 Clustering analysis of expression pattern of pan‐cancer TME signatures

The categorizing method for pan‐cancer TME patterns and 29 sets of gene expression signatures describing pan‐cancer TME characteristics are referred to in Bagaev et al.'s [54] Cancer Cell publication and collected from its corresponding supplementary files. We first calculated signature enrichment scores through GSVA. Then the GSVA scores were robustly standardized (median‐centered and scaled by median absolute deviation) for all the patients. By using the consensusclusterplus R package, we applied an unsupervised clustering algorithm to analyze the standardized GSVA scores of TME signatures in 96 OS patients. The *K*‐means clustering algorithm was used and resampling was set to 1000 repetitions. An analysis of t‐distributed stochastic neighbor embedding (t‐SNE) by using the rtsne r package was further conducted and visualized on a 3D map with the scatterplot3d package of R [55, 56].

### 2.12 Comprehensive evaluation of predictive capacity on response to immunotherapy

To evaluate the predictive capacity of IMP‐associated signature scoring model on response to immunotherapy, we performed comprehensive exploration on two independent approaches: (a) TIDE, Tumor Immune Dysfunction and Exclusion, a computational algorithm to evaluate the potential of tumor immune escape and response to ICI therapy [57]. In our study, normalized gene expression data (VST) was scaled (*z*‐score) for the analysis of the TIDE algorithm. (b) TIS, an 18‐gene T‐cell inflamed signature developed by NanoString Technologies (Seattle, WA) which quantitatively predicts the response to anti‐PD‐1 therapy [58]. We calculated the TIS scores by averaging the normalized gene expression data of the included 18 genes. In addition, we performed exploration on an external clinical dataset with immunotherapy. The IMvigor210 cohort with anti‐PD‐L1 therapy was analyzed in the study on the association of the IMP‐associated signature scoring model and ICI therapy response [59]. The full dataset of the IMvigor210 cohort, including the gene expression data and clinical information, was obtained from http://research‐pub.gene.com/IMvigor210CoreBiologies.

### 2.13 Statistical analysis and visualization

Statistical tests in this study were conducted via the r software (v. 4.0.3, https://www.r‐project.org/). To compare continuous variables, we applied the Wilcoxon test for two groups and a Kruskal–Wallis test for three or more groups. Categorical data were tested by the chi‐square test. The Kaplan–Meier method using the log‐rank test and Cox proportional hazards regression were applied in survival analysis. A statistical test was considered statistically significance at two‐sided *P* < 0.05. We applied the ggplot2 r package for data visualization [60].

## 3 Results

### 3.1 Identification of immune‐related DNA methylation patterns (IMPs) that associates with prognosis of OS patients

We downloaded clinical information and DNA methylation profiles of all OS tissue patients from TARGET and normalized the methylation beta value matrix via the champ R package. A total of 84 patients were matched with DNA methylation profiles and clinical information. We found that a methylation level of 39,335 CpG sites were associated with OS patients' overall survival by univariate Cox regression analysis. In order to explore the immune‐related DNA methylation patterns (IMPs) in OS, we curated an immunologic signature gene set, which included 20,837 genes through combining two previously described databases: ImmuneSigDB and Immport. Referring to the Illumina HumanMethylation450k annotation file, we obtained the target gene of each methylation probe according to the UCSC genome annotation, and further identified 25,924 out of the 39,335 CpG sites that were subject to the immunologic signature gene sets. The methylation beta value matrix of these immunologic CpG sites were extracted for an unsupervised consensus clustering (Table S2).

**Fig. 1:**
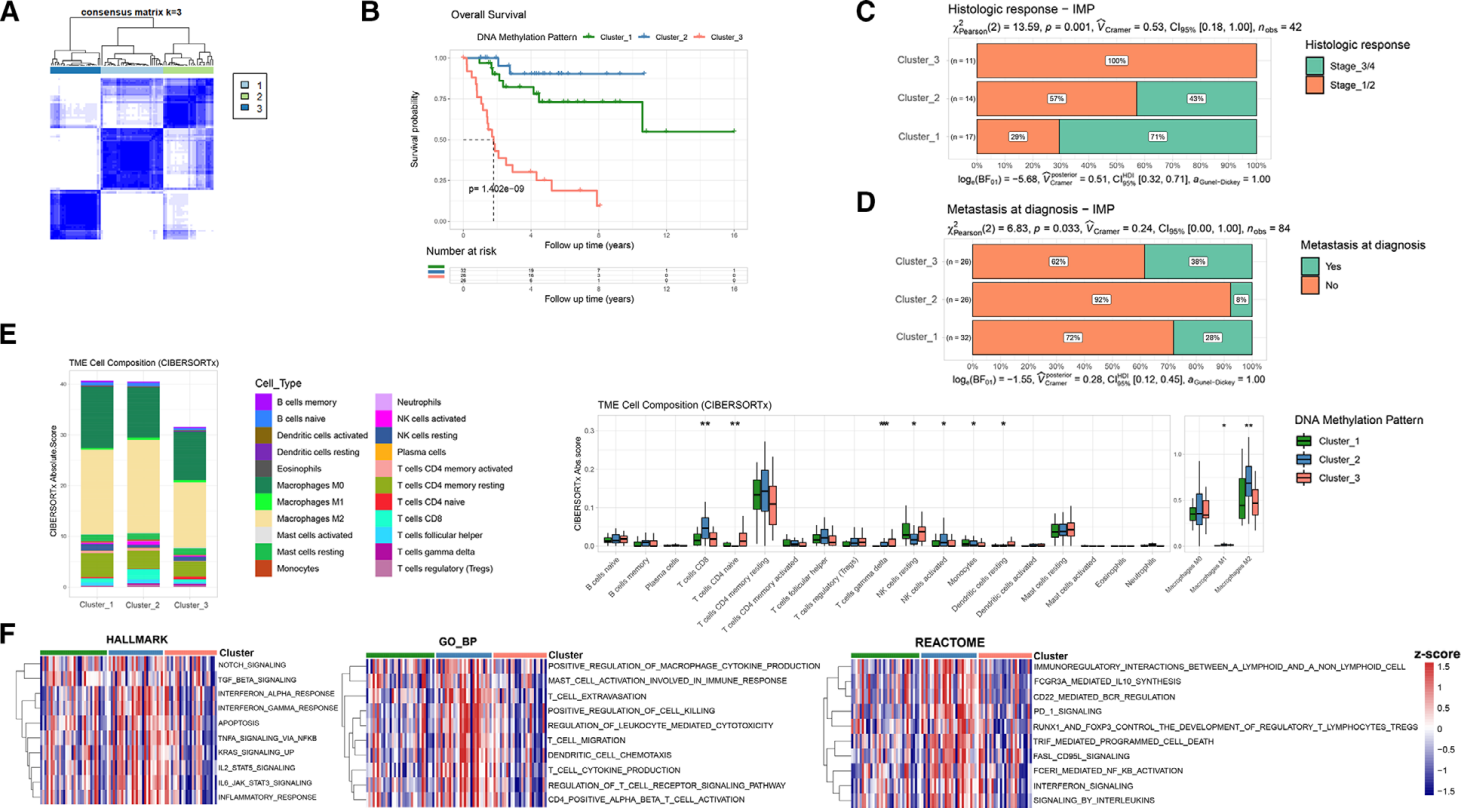
Identification of immune‐related methylation patterns (IMPs) in osteosarcoma and immune infiltration characteristics of different IMPs. (A) An unsupervised consensus clustering (*k* = 3) of Methylation beta value matrix of these immune‐related CpG sites in the TARGET OS cohort. (B) Kaplan–Meier analysis reveals distinct overall survival of the three IMPs (log‐rank test, *n* = 84). (C) Histological response to chemotherapy (Stages 1/2 and 3/4) of patients with OS among three IMPs (chi‐square test, *n* = 42). (D) Metastasis at diagnosis of patients with OS among three IMPs (chi‐square test, *n* = 84). (E) Relative proportion of immune infiltrating cells patients with OS of three IMPs and infiltrating scores of each type of immune infiltrating cells analyzed by CIBERSORTx (Kruskal–Wallis test, *n* = 83). Boxplot displaying the data distribution: the lower and upper hinges represent the first and third quartiles, the upper/lower whiskers extend from the corresponding hinges to the largest/smallest values at most 1.5 * interquartile range from the hinges. Data beyond the end of the whiskers are considered outliers and are not shown. (F) Heatmaps of GSVA enrichment scores (*z*‐score) of hallmark, GO biological process and Reactome pathway gene sets in relation to TME in different IMPs.

As shown in Fig. 1A and Fig. S1A,B, we categorized OS patients into three clusters with a reasonable number of patients in each cluster (*n* = 32, 26 and 26 in clusters 1, 2, and 3, respectively. Average silhouette width: 0.71). To determine the association of IMP and prognosis of OS patients, we performed Kaplan–Meier survival analysis and obtained a *P* value of 1.402e‐09 from the log‐rank test, indicating that patients of different IMPs had distinct overall survival probability. As shown in Fig. 1B, patients in cluster_2 had a satisfactory prognosis. However, the prognosis of patients in cluster_3 was extremely unfavorable, and the overall survival probability drops to 50% in the 2‐year follow‐up and 25% in the 5‐year follow‐up. Furthermore, we compared the clinicopathological characteristics of OS patients (Fig. 1C,D, and Fig. S1C), with no obvious alterations of age, gender, or race among three IMPs. Notably, patients of cluster_3 were observed with an unsatisfactory histological response to chemotherapy and a higher metastasis rate. In order to confirm the robustness of IMP, we performed validation on two independent DNA methylation cohorts (E‐MTAB‐9875 and E‐MTAB‐7263) by extracting the same immunologic CpG sites and conducted unsupervised consensus clustering (Table S2). For the E‐MTAB‐9875 cohort, in order to avoid nonbiological variation, a small number of OS patients tested by 450K BeadChip were excluded. A total of 219 OS patients tested by the EPIC BeadChip were used for clustering. We observed a significantly different distribution of tumor differential grades among three IMPs (*P* = 0.001) (Fig. S1D–F). For the E‐MTAB‐7263 cohort, all 102 chondrosarcoma patients were clustered into three IMPs and we found that a distinct prognosis existed among three IMPs through the Kaplan–Meier survival analysis (*P* = 3.05e−11) (Fig. S1G–I), in which patients of cluster 3 have an extremely poor overall survival.

### 3.2 Immune infiltration characteristics of different IMPs

To comprehensively evaluate tumor immune microenvironment characteristics of the three IMPs, we extracted the transcriptomic profile of each sample and applied two independent deconvolution algorithms: CIBERSORTx and quanTIseq, to calculate specific immune cells composition in the TME (Table S3). The results from both analytic approaches presented significantly different immune infiltration features in the TME of three IMPs, indicating that the patients in different IMPs were of different immune cells infiltration landscapes.

As shown in Fig. 1E, CD8^+^ T‐cell infiltration was significantly higher in cluster_2 patients, while T cells CD4 naive infiltration was significantly higher in cluster_3 patients compared to the other two clusters. Cytotoxic CD8^+^ T cells can recognize tumor‐specific (neoantigens) or tumor‐associated antigens and exert an antitumor function by releasing perforin and granzymes, etc. [61]. In addition, patients of cluster_2 were found to have an obviously higher level of activated NK cells, and a lower level of resting NK cells. Patients of cluster_3 were found to have a relatively higher infiltration level of γδT cells, which are known to promote cancer progression by producing IL‐17 or facilitating the ability on myeloid derived suppressor cells in TME [62, 63], although antitumorigenic effects of γδT cells through various mechanisms have also been reported [64, 65, 66]. The quanTIseq analysis revealed apparently lower infiltration of macrophages, neutrophils, CD4^+^ T cells, and higher infiltration of B cells, DCs in cluster_3 (Fig. S1J). The pro‐ and antiinflammatory macrophages may have complex crosstalk and biological roles along with the varying TME status [67]. The CD4^+^ T cells are associated with the enhanced tumor immune response [68]. Nonetheless, the controversial protumor or antitumor roles of B cells [69], neutrophils [70] and DCs [71] have been reported and may be affected by the tumor immune microenvironment in different cancers. Moreover, we applied the xCell algorithm, a practical method for inferring multiple immune and stromal cell types based on ssGSEA, to evaluate the overall TME composition in different clusters (Table S3). We found a higher TME score and immune score in cluster_2, while no significant difference of stomal score was found among the three clusters (Fig. S1K).

In order to assess whether the distribution of immune cells coincide with our finding that a distinct prognosis exists among IMPs, we carried out the Kaplan–Meier survival analysis to examine potentially beneficial or harmful significance of the infiltration level on OS patients' prognosis of each cell type. The “surv_cutpoint” function of the “survminer” r package was used to determine the optimal cutoff point of the CIBERSORTx absolute scores (the minimal proportion of observations per group was set to 20% to avoid the occurrence of too few individuals in a certain group). As shown in Fig. S1L, we observed that the high infiltration levels of T cells CD4 memory resting, T cells follicular helper, T cells CD8, Monocytes, and Macrophages M2 were beneficial to OS patients' overall survival, while that of T cells CD4 naive, NK cells resting, and Dendritic cells resting could be harmful. Together, T cells CD8, T cells CD4 naive, NK cells resting, Monocytes, Dendritic cells resting and Macrophages M2 infiltrations were shown to be consistent in the distribution among IMPs and the potential impact on survival.

The gene set variation analysis (GSVA) analysis indicated that some cancer‐related hallmarks and pathways varied among the IMPs, as shown in the heatmaps of GSVA enrichment scores such as: apoptosis, Notch signaling and KRAS signaling, and immune biological processes including interleukins‐, interferons‐, TNFα‐ and TNFβ‐signaling, CD4^+^ αβT cells activation, DCs chemotaxis and leukocytes mediated cytotoxicity, and PD‐1 signaling pathways (Table S3 and Fig. 1F). Taken together, our results demonstrated that elevated immune infiltration, enhanced cytotoxic potential, and activated antitumor immune response were found in the cluster_2 OS patients, while the cluster_3 presented with immunosuppressive TME status, and hence was associated with better prognosis in the cluster_2 but shorter survival in the cluster_3.

### 3.3 Characteristics of DNA methylation and gene expression in IMPs

**Fig. 2:**
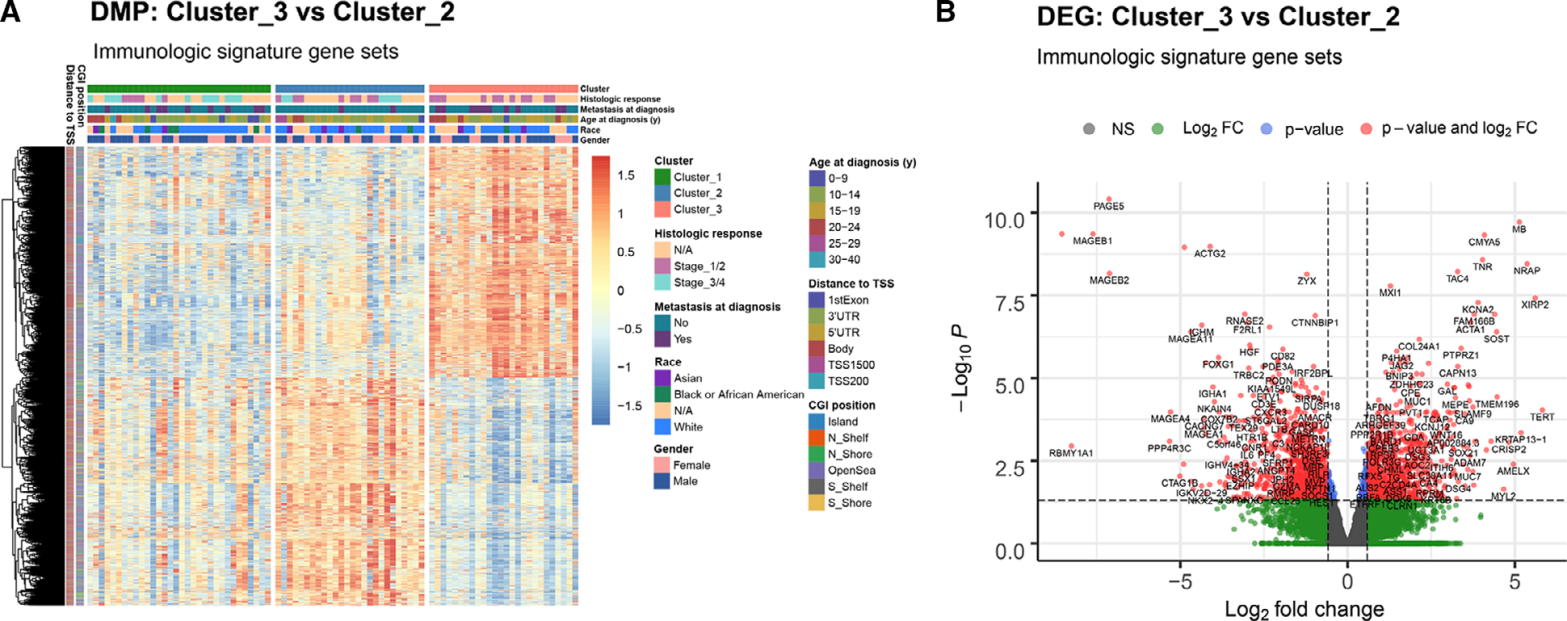
Characteristics of DNA methylation and gene expression of IMPs. (A) A heatmap of methylation levels (*z*‐score) of differentially methylated probes (immunologic signature gene sets) between cluster 3 and cluster 2 of IMP. (B) A volcano plot differentially expressed genes (immunologic signature gene sets) between cluster 3 and cluster 2 of IMP (Wald test implemented in the deseq2, *n* = 52).

With regard to the most distinct overall survival and TME‐infiltrating cells composition between cluster_2 and 3, we performed differentially methylated probes (DMPs) and differentially expressed genes (DEGs) analyses (Table S4). Compared to cluster_2, a total of 32,805 DMPs (15,410 hypomethylated and 17,395 hypermethylated) and 2747 DEGs (1516 upregulated and 1231 downregulated) were identified in cluster_3 (Fig. S2A,B). Among them, 8176 DMPs and 1910 DEGs were found to form the immunologic signature gene sets (Fig. 2A,B).

To study the regulatory effect of DNA methylation on gene expression of the 1910 immunologic DEGs, we performed Pearson correlation analysis to inspect both cis‐ and transregulation. For the cis‐regulation effect, where gene expression is usually colocated with the DNA methylation level of its promoter region, we found methylation levels of 2047 CpG sites corresponding to 754 genes, that were significantly correlated with gene expression levels (1309 negative and 738 positive correlations), consistent with the notion that cis‐regulation usually implies a negative correlation between promoter methylation and gene expression. The transregulation effect, where the methylation level at the promoter region of one gene is correlated with expression level of other genes, was analyzed on the DEGs and DMPs among IMP cluster_2 and 3. We found most DEGs (1638 out of 1910) were transregulated by immune‐related DMPs (Table S4).

### 3.4 Construction of an IMP*‐*associated signature scoring model

As the above results indicated that possible relationships might be extrapolated between clinical outcomes and immune phenotypes among IMPs, we sought to build a signature scoring model to quantitatively evaluate the IMPs and assess the prognosis of individual OS patients. We included a total of 96 patients with matched RNA‐Seq data and clinical follow‐up data from TARGET as the training cohort, and used an independent OS dataset from GEO (GSE21257, *n* = 53) as the testing cohort for validation. We analyzed the 1910 immunologic DEGs between IMPs cluster_2 and 3 by the univariate Cox regression and identified the expression levels of 662 genes were associated with overall survival.

**Fig. 3:**
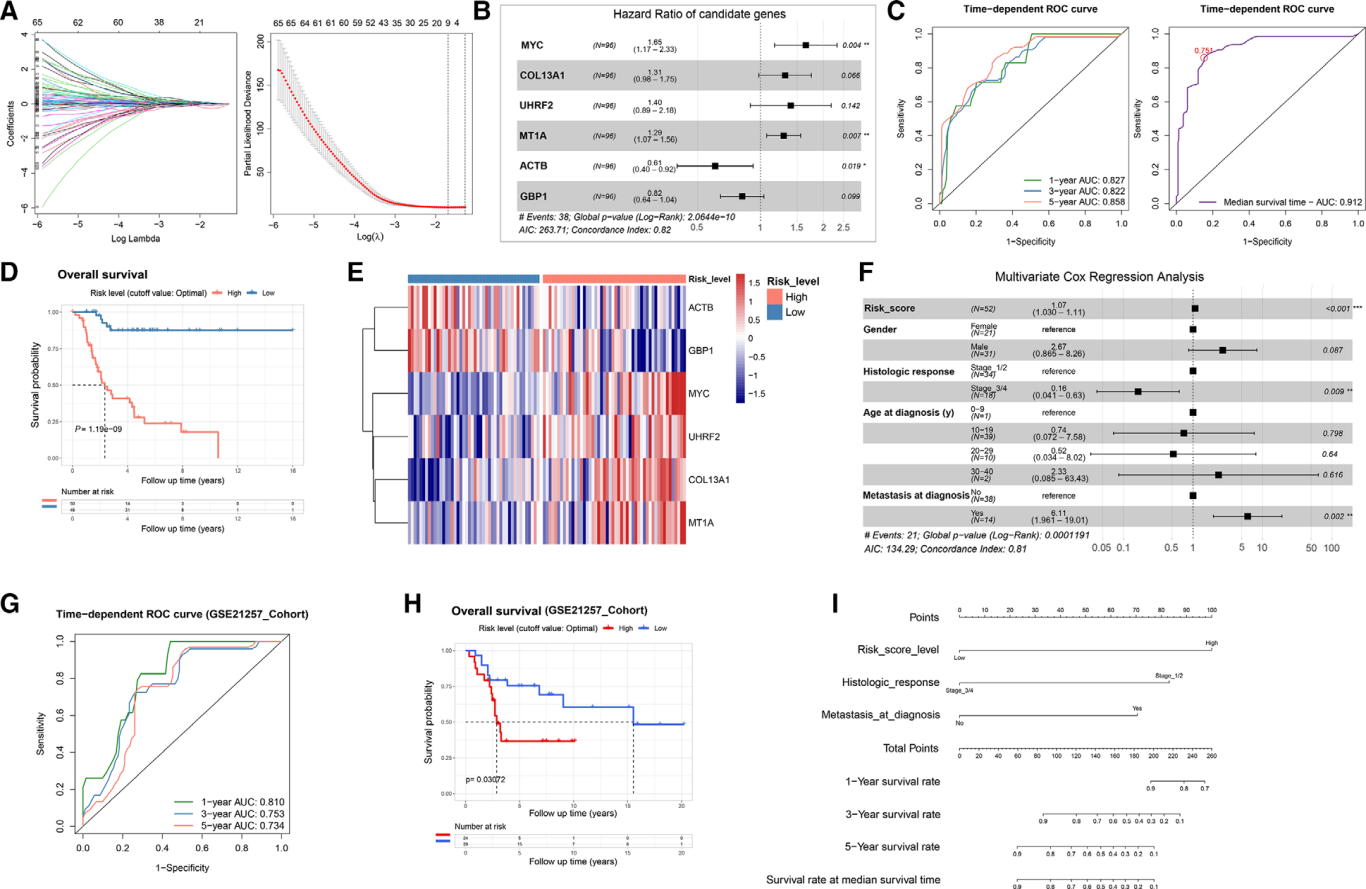
Construction and validation of an IMP‐associated signature scoring model in osteosarcoma. (A) A total of nine candidates were screened through Lasso cox regression analysis of genes identified by univariate cox regression analysis. Left: Variable and coefficient profiles in the LASSO Cox regression model. Right: Tenfold cross‐validation of the LASSO Cox regression model for the tuning parameter selection. The horizontal axis represents the log (lambda) value, and the vertical axis represents partial likelihood deviance. The red dots represent partial likelihood deviance for a tuning parameter, and error bars represent standard errors. (B) A forest plot of the six‐gene model determined by the stepwise regression model using the Akaike Information Criterion (AIC) method. The black dots represent hazard ratios (HRs), and error bars represent 95% confidence intervals (CIs) (multivariate Cox regression). (C) Time‐dependent ROC curve analysis of the IMP‐associated signature scoring model (IMP Risk scores) for predicting the overall survival probability of 1‐, 3‐, 5‐year and median‐survival time of patients with OS in the TARGET cohort. (D) Kaplan–Meier analysis on overall survival of high‐ and low‐IMP Risk groups divided by the optimal cutoff point (log‐rank test, *n* = 96). (E) A heatmap of the six‐gene signature expression (*z*‐score) of high‐ and low‐IMP Risk groups. (F) A forest plot of multivariate Cox regression analysis of IMP‐associated signature scores and other four clinicopathological characteristics. The black dots represent HRs, and error bars represent 95% CIs (multivariate Cox regression). (G, H) Valuation of the IMP‐associated signature scoring model on an external OS cohort: time‐dependent ROC curve analysis of the IMP‐associated signature scoring model (IMP Risk scores) for predicting the survival probability of 1‐, 3‐, 5‐year follow‐up time and Kaplan–Meier analysis of high‐ and low‐IMP Risk groups (divided by optimal cutoff point) of patients in the GSE21257 cohort on overall survival (log‐rank test, *n* = 53). (I) A nomogram comprising IMP‐associated signature scores, histologic response to chemotherapy, and metastasis state for predicting the overall survival probability of patients with OS.

Moreover, a Lasso penalized Cox regression analysis identified nine genes for constructing the prognostic risk model. Based on the method of stepwise regression using the Akaike's information criterion (AIC) method, we constructed a six‐gene prognostic risk scoring model with most optimal candidates (Fig. 3A,B and Table S5). An IMP_Risk score = expression level of *MYC* * 0.4998 + expression level of *COL13A1* * (0.2715) + expression level of *UHRF2* * (0.3338) + expression level of *MT1A* * (0.2558) + expression level of *ACTB* * (−0.4997) + expression level of *GBP1* * (−0.2012). The concordance index (*C*‐index) of the risk score model was 0.82 (*P* = 2.06e‐10). As shown in the time‐dependent ROC curves (Fig. 3C), AUCs were 0.827, 0.822, 0.858 for the 1, 3, 5‐year overall survival prediction by IMP_Risk scores, respectively. For the overall median survival time of the training cohort (3862 days, Fig. S2C), the AUC was calculated to be 0.912 and we defined 0.751 of the IMP_Risk score as the cutoff value for dividing high‐ and low‐IMP Risk groups. As shown in Fig. 3D and Fig. S2D, Kaplan–Meier survival analysis showed high‐IMP_Risk patients had significantly shorter overall survival (log‐rank *P* = 1.19e‐9). The distribution of the risk score, survival status, and the six‐gene expression profile between high‐ and low‐risk score groups is shown in Fig. S2E and Fig. 3E.

In order to confirm whether the IMP_Risk score was independent with other clinical features, patients with available clinicopathologic parameters were included for a univariate Cox regression analysis. We retained parameters with *P*‐values less than 0.1 in a multivariate Cox regression analysis. As shown in Table S5 and Fig. 3F, the IMP_Risk score, histologic response, and metastasis at diagnosis were identified as independent prognostic factors of overall survival. We also verified the good predictive capacity on relapse‐free survival of the IMP‐associated signature scoring model by using time‐dependent ROC analysis for the 1, 3, 5‐year overall survival and Kaplan–Meier survival analyses (Fig. S2F).

A total of 53 OS patients from GSE21257 with matched clinical follow up and microarray data were used for validation of the IMP‐associated signature scoring model. The IMP_Risk score of each patient was calculated using the same formula with normalized gene expression data. Through the time‐dependent ROC analysis on overall median survival time (5670 days, Fig. S2G), we determined 5.95 of the IMP_Risk score as the cutoff point for dividing high‐ and low‐IMP_Risk groups (Fig. S2H). As shown in Fig. 3G,H and Fig. S2I, both time‐dependent ROC and Kaplan–Meier survival analyses were performed likewise, and we found that the IMP‐associated signature scoring model maintained excellent prognosis predictive capacity on both overall survival and metastasis‐free survival. Therefore, the results of both the training and testing cohorts have demonstrated that the IMP‐associated signature scoring model can function as an excellent model for predicting short‐term or long‐term overall survival and relapse/metastasis‐free survival in OS patients, which would benefit our decision‐making on therapeutic strategies and/or prediction on long‐term prognosis/clinical outcomes of OS patients.

For the TARGET‐OS dataset, we further integrated the IMP_Risk score, histologic response, and metastasis at diagnosis to construct a nomogram, and the results showed that the IMP_Risk score was a major risk contributor (Fig. 3I). As shown in the calibration curves, the nomogram offered an ideal predictive accuracy for OS patients' overall survival (Fig. S2J). In addition, we conducted a decision curve analysis (DCA) and showed that the IMP_Risk score had an evidently higher net benefit, compared to histologic response and metastasis at diagnosis (Fig. S2K). Taken together, these results indicated that the IMP‐associated signature scoring model and nomogram provided excellent capacity and consistency for overall survival prediction.

### 3.5 Molecular characteristics associated with IMP‐associated signature scores

**Fig. 4:**
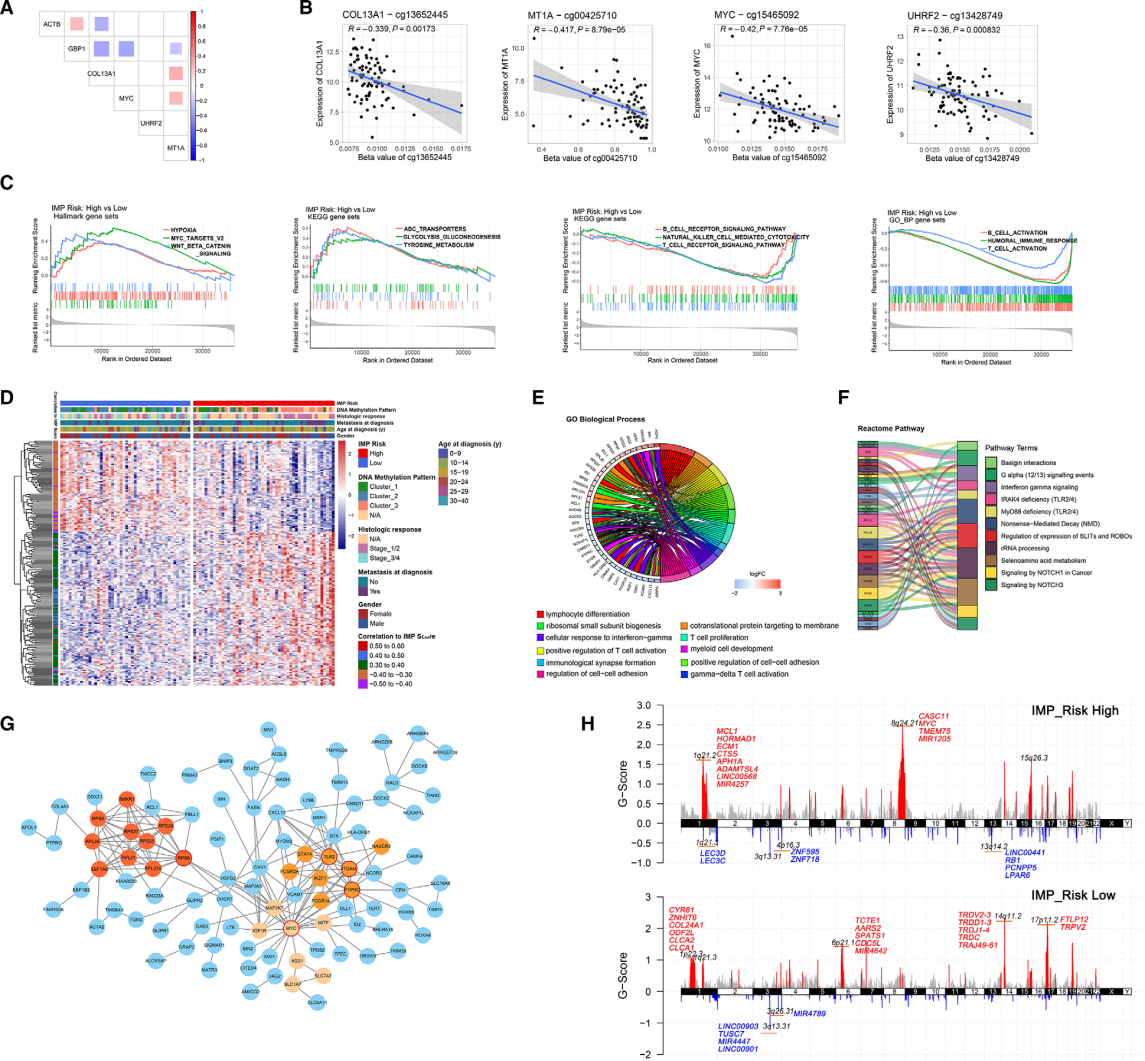
Integrative analysis of molecular characteristics of osteosarcoma and IMP‐associated signature scoring model. (A) Co‐occurrence of the six‐gene signatures in IMP‐associated signature scoring model. (B) Correlation analysis of gene expression level and DNA methylation level at the promoter region of the genes in IMP‐associated signature scoring model (Pearson correlation test, *n* = 83). Gray zone represents the 95% CI for prediction from a linear model. (C) GSEA plots of altered hallmarks, GO biological processes, and KEGG pathways gene sets between high‐ and low‐IMP Risk subgroups. (D) A heatmap for the expression (*z*‐score) of IMP Risk scores associated immune genes. (E) A circular plot for the overrepresentative analysis in GO biological processes of the IMP Risk scores associated immune genes. (F) A Sankey plot for overrepresentative analysis in Reactome pathways of the IMP Risk scores associated immune genes. (G) PPI analysis of the IMP Risk scores associated immune genes. The three most correlated subclusters are highlighted by varied color and Top‐5 hub genes are marked with a red circular border. (H) Recurrent somatic CNV identified by GISTIC 2.0 in high‐ and low‐IMP Risk subgroups (*n* = 42 and 42, respectively).

Using the Pearson correlation analysis, either positive or negative coexpression was analyzed among the six genes included in the IMP‐associated signature scoring model (Fig. 4A). We found the expression levels of *MYC*, *COL13A1*, *UHRF2*, and *MT1A* were negatively correlated with methylation levels of specific promoter CpG sites (Fig. 4B, Fig. S3A, and Table S6). A total of 746 upregulated and 876 downregulated DEGs were found between high‐ and low‐IMP_Risk groups, in which 474 upregulated and 579 downregulated genes were associated with immunologic signature gene sets (Fig. S3B). Through the preranked GSEA, we found that several biological processes and pathways, such as cytokine or chemokine signaling and humoral or cellular immune response, were significantly activated in the low‐IMP_Risk group, while hypoxia, *MYC* targets, Wnt/β‐catenin signaling, tyrosine metabolism, and other tumorigenesis‐related pathways were more enriched in the high‐IMP_Risk group (Fig. 4C, Fig. S3C, and Table S6).

To gain more insight into molecular characteristics associated with IMP‐associated signature scores, 218 immune DEGs were identified as an IMP_Risk score highly associating genes through Pearson correlation analysis (absolute Pearson correlation coefficient ≥ 0.3 and FDR < 0.05, Table S6). A heatmap revealed that the expression of 218 immune DEGs correlated with IMP_Risk scores (Fig. 4D). Overrepresentation analysis identified the enriched biological functions and pathways of these genes, such as IFN‐γ signaling, T‐cell proliferation, and activation, cell–cell adhesion, SLIT/ROBO regulation, and NOTCH1/3 signaling pathways (Fig. 4E,F). In addition, we built a PPI network via the STRING database and established prospective protein–protein interactions among these genes. As shown in Fig. 4G, we identified top‐5 hub genes and top‐3 hub clusters of the global PPI network through cytoscape mcode and hubba plugins.

Furthermore, we evaluated the divergence of somatic copy number alternations (SCNAs) between high‐ and low‐risk OS patients by using GISTIC 2.0, and identified specific chromosome regions with significant amplification or deletion between different IMP_Risk groups (Table S6). As shown in Fig. 4H, amplifications on chromosomes 1 and 8 accompanied with deletions on chromosome 13 were enriched in the high‐risk group, while amplifications on chromosomes 14 and 17 accompanied with deletions on chromosome 3 were enriched in the low‐risk group. Focal amplification peaks, including the well‐studied cancer‐driven gene *MYC* (8q.24.21) and several antiapoptotic genes (*MCL1*, *HORMAD1*, and *ECM1* on 1q21.2), were identified in the high‐risk patients, along with a focal deletion peak at 13q14.2. It is noteworthy that focal amplification peaks including multiple TCR‐related genes (14q11.2 and 17p11.2) were identified in the low‐IMP_Risk group.

### 3.6 IMP‐associated signature scores involved in pharmacogenomic interactions

**Fig. 5:**
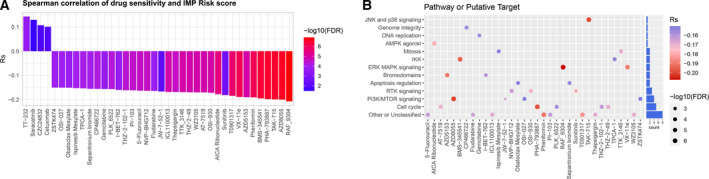
The relationship between IMP‐associated signature scores and drug sensitivity. (A) The correlation between IMP Risk scores and drug sensitivity (AUC values of GDSC) examined by the Spearman analysis (sensitivity data of 518 drugs and gene expression profiles of 1014 cell lines). Correlation to drug response is indicated by Spearman coefficient with *R*_S_ > 0 for resistance and *R*_S_ < 0 for sensitivity. (B) Putative targets or functional pathways of the drugs that are sensitivity to the IMP Risk scores.

To further understand the potential association of the IMP_Risk score and drug response, we applied the Spearman correlation analysis to identify significantly correlated pairs between the IMP_Risk score and drug sensitivity based on the Genomics of Drug Sensitivity in Cancer (GDSC) database, which documented the transcriptional profile and drug sensitivity data on about 1000 cancer cell lines (Fig. 5A, Table S6). We identified 34 pairs in which drug sensitivity was correlated with the IMP_Risk score, including PI3K/MTOR inhibitor AZD8055 (*R*_S_ = −0.20, *P* = 8.80e‐10), CDK inhibitor PHA‐793887 (*R*_S_ = −0.20, *P* = 2.27e‐09), and RTK inhibitor sunitinib (*R*_S_ = −0.18, *P* = 0.00035). We also identified four pairs in which drug resistance was correlated with the IMP_Risk score, including Src and Abl kinase inhibitor saracatinib (*R*_S_ = 0.13, *P* = 0.01) and EGFR inhibitor cetuximab (*R*_S_ = 0.10, *P* = 0.003). In addition, we interrogated putative targets and signaling pathways of the drugs sensitive to the IMP_Risk score, and found that most of them targeted JNK/p38, ERK/MAPK, PI3K/mTOR, and cell cycle signaling pathways (Fig. 5B).

### 3.7 Association between IMP‐associated signature scores and TME patterns in OS

**Fig. 6:**
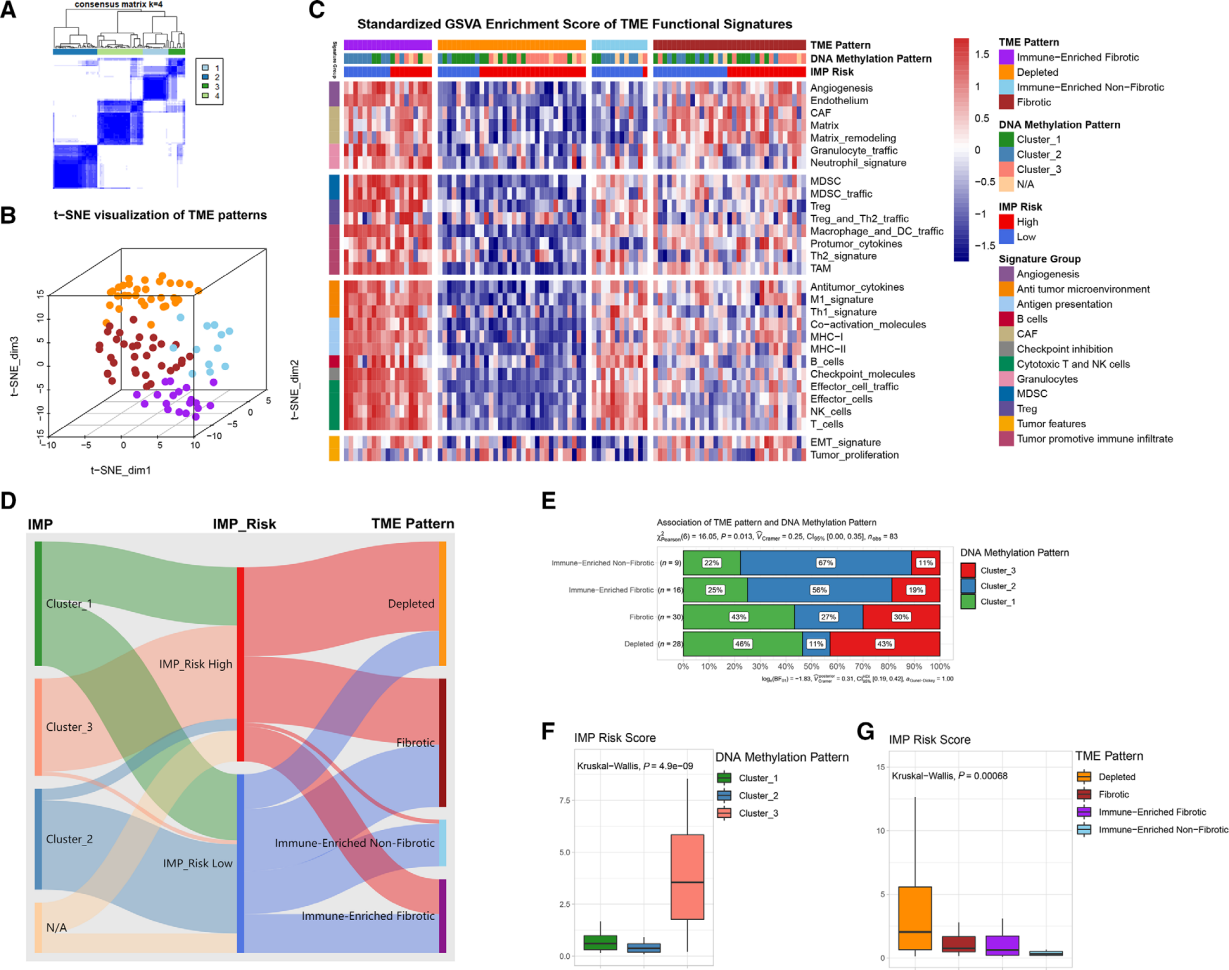
IMP‐associated signature scores reveal distinct tumor microenvironment patterns in osteosarcoma. (A) An unsupervised consensus clustering (*k* = 4) of the robustly standardized GSVA enrichment scores of TME‐pattern signature gene sets in TARGET OS cohort. (B) A 3D t‐sne distribution of OS patients corresponding to each TME pattern based on unsupervised consensus clustering. (C) A heatmap of the robustly standardized GSVA enrichment scores for OS patients assigned into four distinct TME patterns based on unsupervised consensus clustering. (D) A Sankey plot that displays the affiliation among IMPs, IMP Risk levels, and TME patterns. (E) The detailed proportions of IMPs of each TME pattern (chi‐square test, *n* = 83). (F, G) Boxplots showing the distribution of IMP Risk scores among different IMPs and TME patterns, respectively (Kruskal–Wallis test, *n* = 83 and 96).

To investigate the complex role of TME in mediating cancer progression and metastasis, Bagaev et al. [54] defined four distinct pan‐cancer TME subtypes, namely, immune‐enriched, fibrotic (IE/F); immune‐enriched, nonfibrotic (IE); fibrotic (F); and depleted (D). Among the four TME patterns, patients of the two immune‐enriched patterns had relative better prognosis, especially for those of the TME_IE pattern in specific types of cancer. However, patients of the TME_D pattern had consistently poor prognosis across pan‐cancer datasets. Similarly, we utilized an unsupervised clustering method to assign the TARGET OS patients into four groups by using robustly standardized GSVA enrichment scores of the 29 functional gene expression signatures (F^GES^) sets (Fig. S4A and Fig. 6A, Table S7). As shown in Fig. 6B, t‐SNE analysis showed the apparent distribution of GSVA results among the four TME patterns. Detailed standardized GSVA enrichment scores of each OS sample, as shown in a heatmap, revealed distinct F^GES^ characteristics among the four TME patterns (Fig. 6C).

Collectively, three IMP clusters, high/low IMP‐associated signature scoring risk levels, and four TME patterns displayed significant concordant relationships among OS patients in TARGET (Fig. 6D–G). For example, the high‐IMP_Risk group contained most of IMP cluster_3 and a higher proportion of the TME_D pattern, whereas IMP cluster_2 and TME_IE pattern were more enriched in the low‐IMP_Risk group. Consistent with previously published results, IMP cluster_2 represented 67% and 56% of the TME_IE and IE/F pattern with better prognosis, while the TME_D pattern with poor prognosis was more enriched in IMP cluster_3, which was predominantly associated with high‐IMP_Risk scores.

### 3.8 Prediction of response to immunotherapy using the IMP*‐*associated signature scoring model

As we demonstrated that IMP‐associated signature scores were associated with TME patterns in OS, we sought to study whether the IMP‐associated signature scoring model could predict OS patients' response to immunotherapy. We utilized the Tumor Immune Dysfunction and Exclusion (TIDE) module to assess the potential clinical efficacy of immunotherapy on patients of different IMP_Risk groups. The TIDE algorithm evaluates the expression signatures of T‐cell dysfunction and T‐cell exclusion to assess tumor immune evasion and integrate them into a total TIDE score. A higher TIDE prediction score represents a higher potential for immune evasion, indicating that the patients are less likely to benefit from ICI therapy. In addition, the TIDE module analyzes multiple features to estimate tumor immune evasion, such as correlation with the MDSC, TAM or CAF signatures.

**Fig. 7:**
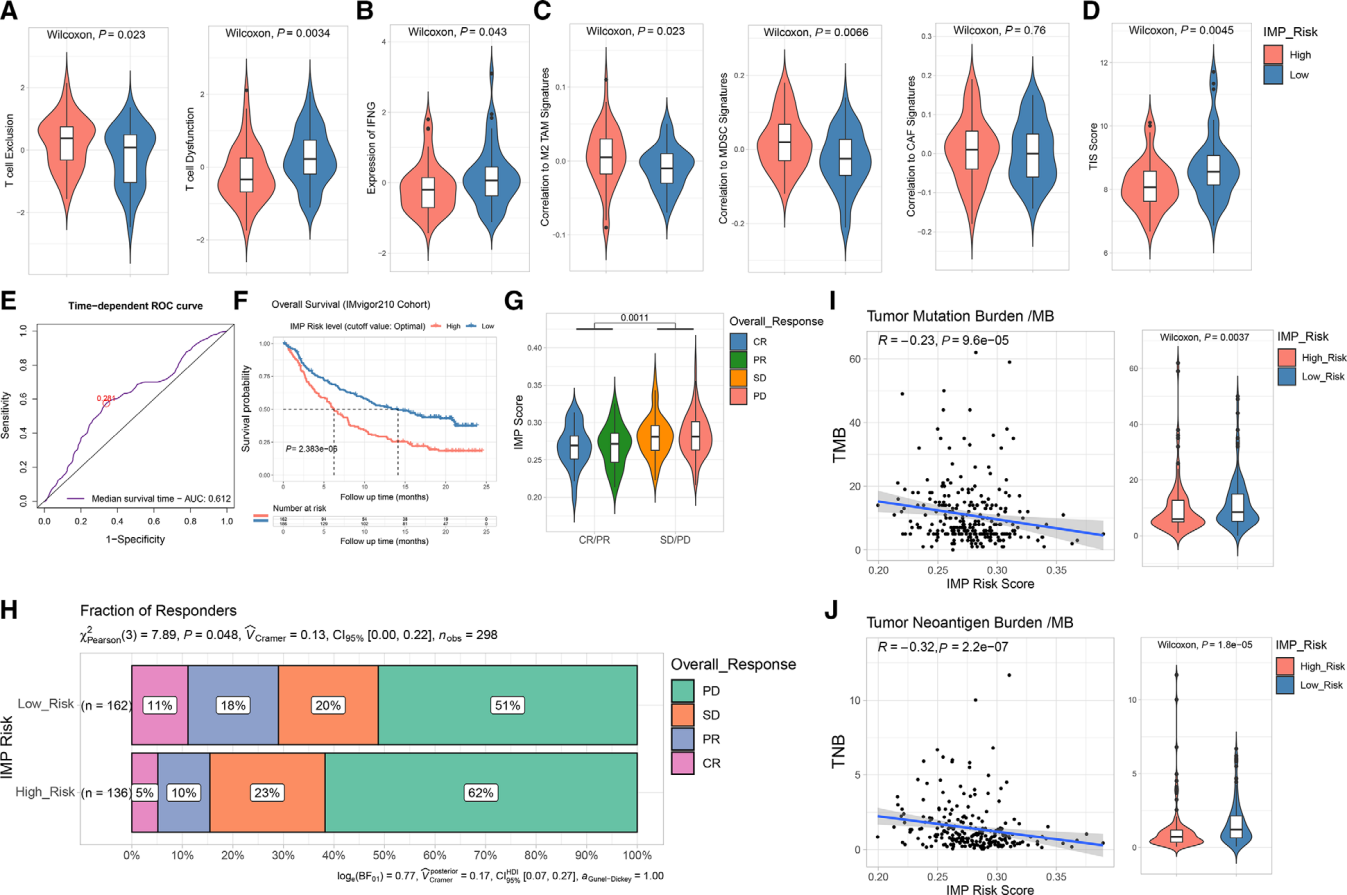
Predictive value of IMP‐associated signature scoring model on response to immunotherapy. (A) Violin and boxplots showing T‐cell exclusion and dysfunction of high‐ and low‐IMP Risk subgroups evaluated by TIDE (Wilcoxon test, *n* = 96). (B) Violin and boxplots showing IFNG signature expression of high‐ and low‐IMP Risk subgroups evaluated by TIDE (Wilcoxon test, *n* = 96). (C) Violin and boxplots showing correlations to expressions of M2 TAM, MDSC, and CAF‐associated signatures of high‐ and low‐IMP Risk subgroups evaluated by TIDE (Wilcoxon test, *n* = 96). (D) Violin and boxplots showing TIS (T‐cell inflamed signature) scores of high‐ and low‐IMP Risk subgroups (Wilcoxon test, *n* = 96). (E) A time‐dependent ROC curve analysis of the IMP‐associated signature scoring model for predicting the overall survival probability on median‐survival time of the IMvigor210 cohort (*n* = 348). (F) Kaplan–Meier analysis on overall survival of high‐ and low‐IMP Risk groups divided by optimal cutoff point in the IMvigor210 cohort (log‐rank test, *n* = 348). (G) Violin and boxplots showing distribution of IMP Risk scores of different overall responsive subgroups in the IMvigor210 cohort (CR, complete response; PR, partial response; SD, stable disease; PD, progressive disease) (Kruskal–Wallis test, *n* = 298). (H) Proportions of overall responsive subgroups corresponding to high‐ and low‐IMP Risk levels (chi‐square test, *n* = 298). (I, J) Spearman correlation analysis and Wilcoxon test for tumor mutation burden (TMB) and tumor neoantigen burden (TNB) on IMP Risk scores and levels in the IMvigor210 cohort. Data distribution displayed by violin plots. Gray zone in the correlation plots represent the 95% CI for prediction from a linear model (*n* = 272 and 245, respectively).

Using the TARGET OS datasets, we did not find any significant differences of overall TIDE scores between high‐ and low‐risk groups (Fig. S4B). Nonetheless, we found relatively higher T‐cell exclusion scores, but lower T‐cell dysfunction scores in the high‐risk group (Fig. 7A). To our knowledge, a lower T‐cell dysfunction score in the high‐risk group could result from depleted T cells infiltration. For other features produced by TIDE, we found that the lower expression of a IFNG signature was correlated with M2 TAM, MDSCs, and CAFs signatures of the high‐risk group (Fig. 7B,C). A higher proportion of CTL was associated with the low‐risk group (Fig. S4C). Moreover, we evaluated the T‐cell inflamed signature (TIS) scores and found relatively lower TIS scores in the high‐risk group (Fig. 7D and Table S7). Taken together, these results indicated that OS patients in the high‐risk group might have a higher potential of immunosuppressive TME status and worse response to ICI therapy.

Lastly, we used the IMvigor210 cohort, PD‐L1 therapy follow‐up datasets, to assess the capability of IMP_Risk scores in predicting the ICI therapy response, and found that patients of the low‐IMP_Risk were provided with significant clinical benefits, better therapeutic responses, and a markedly prolonged overall survival after PD‐L1 therapy (Fig. S4D and Fig. 7E–H, Table S7). Furthermore, we found the tumor mutation burden (TMB) and tumor neoantigen burden (TNB) were significantly higher in the low‐IMP_Risk group (Fig. 7I,J), which may at least partially explain the advantage and the greater benefit of ICI therapy for the low‐IMP_Risk group.

Collectively, our results strongly suggest that the established IMP‐associated signature scoring model may effectively predict the response to ICI therapy and clinical outcomes of OS patients.

## 4 Discussion

Epigenetic processes regulate gene expression without altering the DNA sequence. As a heritable epigenetic mark, DNA methylation is a biological process where a methyl group is transferred to the C5 position of the cytosine of DNA through DNA methyltransferases (DNMTs) [7]. Broad alterations of the DNA methylation have been shown to accompany cancer initiation and development, although certain DNA methylation patterns are cancer‐specific and conserved across individuals [10]. Accumulating studies in the past two decades have revealed the important role of epigenetic therapies in directly killing cancer cells, but also modulating antitumor immune response [72]. In this study we clustered OS patients into three immune methylation patterns (IMPs) based on methylation levels of CpG sites related to immunologic gene sets, and demonstrated distinct prognosis, clinicopathologic characteristics, as well as the immune infiltration landscape in different IMPs.

Based on the relative robustness and tissue‐specific characteristics of DNA methylation, detection of DNA methylation‐related biomarkers has been developed to aid in cancer diagnosis and prognosis prediction. Rosenblum et al. [73] reported the significant correlation between DNA methylation profiles and clinical outcomes of OS patients. Tian et al. [74] identified several methylated DEGs as OS survival‐related genes, which might be helpful in early diagnosis and precision therapy. Deng et al. [9] established a four‐methylated lncRNA signature to predict OS patients' survival. Here, we constructed a robust IMP‐associated signature scoring model, which constitutes six genes: *MYC*, *COL13A1*, *UHRF2*, *MT1A*, *ACTB*, and *GBP1* to quantitatively evaluate IMPs and hence predict the prognosis of individual OS patients. Our IMP‐associated signature scoring model was validated as an independent prognostic factor for OS patients. Furthermore, we developed a nomogram by combining the IMP_Risk score, histologic response to chemotherapy, and metastasis at diagnosis for prognosis prediction, which can be used as a valuable tool to aid our decision‐making for the clinical management of osteosarcoma.

The *c‐MYC* proto‐oncogene encodes a transcription factor and plays a crucial role in tumorigenesis such as proliferation, growth, apoptosis, metabolism, DNA replication, and angiogenesis. Aberrant MYC DNA methylations in multiple myeloma, prostate cancer, and colon cancer are associated with more aggressive cancer progression and metastasis [75, 76, 77]. Collagen type XIII is a transmembrane protein localized in cell–cell and cell–extracellular matrix (ECM) junctions, and was implicated in a tumor suppressor in the development of intestinal lymphomas [78], although Miyake et al. [79] identified the association between *COL13A1* overexpression and increased invasion capability in urothelial cancer. The controversial roles of UHRF2 have been reported in different cancers, as its oncogenic role was reported in colorectal cancer through stabilizing TCF4 mediated Wnt/β‐catenin signaling, while it acted as a negative regulator of epithelial‐mesenchymal transition (EMT) in esophageal squamous cell carcinoma [80, 81]. *MT1A* is a member of metallothionein genes, which are implicated as emerging modulators in immune response [82]. Aberrant *MT1A* gene methylation and expression were associated with glioma progression [83]. Lastly, the roles of guanylate‐binding protein 1 (GBP1) in cancer are seemingly context‐dependent, since its upregulation was associated with decreased progression in breast and colorectal cancer, but increased progression, metastasis, and resistance in ovarian cancer and glioblastoma [84]. Thus, except for *C‐MYC*, the exact functions of five of the six signature genes remain to be fully understood.

To explore molecular characteristics associated with IMPs, we performed multilevel analyses between high‐ and low‐IMP_Risk groups. Through the GSEA, we showed that immune response‐related signaling pathways were significantly activated in the low‐IMP_Risk group, while MYC targets, Wnt/β‐catenin signaling, and other tumorigenesis‐related pathways were more enriched in the high‐IMP_Risk group. The overrepresentation analysis identified IMP_Risk scores‐associated genes were enriched with IFN‐γ signaling, T‐cell proliferation and activation, and NOTCH1/3 signaling pathways. Thus, we speculated that IMP_Risk‐related biomarkers may play a pivotal role in OS tumorigenesis through the aforementioned biological processes, while an intense immune response phenotype may exist in patients of the low‐IMP_Risk group.

The SCNA analysis revealed that high‐IMP_Risk patients had multiple recurrent focal amplification peaks covering genomic regions of *MYC*, *MCL1*, *HORMAD1*, and *ECM1*, while several focal amplification peaks of TCR‐related genes were detected in low‐IMP_Risk OS patients. MCL‐1 is an antiapoptotic BCL‐2 family protein and prevents intrinsic apoptosis by binding and sequestering proapoptotic BCL‐2 family members such as BIM, NOXA, BAX, or BAK. Although OS cell lines remained insensitive to single inhibition of MCL‐1, Kehr et al. [85] reported that dual inhibition of BCL‐XL and MCL‐1, either by inhibitors or siRNA transient silencing, induced potent and rapid apoptosis via the mitochondrial pathway in pediatric solid cancer. Furthermore, based on pharmacogenetic data reported by GDSC, we found that the IMP_Risk score was associated with resistance to drugs targeting Src and Abl kinase and EGFR signaling pathways, while sensitive to drugs targeting JNK/p38, ERK/MAPK, PI3K/mTOR, and cell cycle signaling pathways. These results imply that high‐IMP_Risk OS patients may benefit from PI3K/MTOR inhibitor AZD8055, CDK inhibitor PHA‐793887, and RTK inhibitor sunitinib.

It has been recently highlighted that DNA methylation plays a vital role in reconfiguring the TME and modulates the crosstalk between tumor and stromal, immune cells. Accordingly, patients in IMP cluster_2 had a favorable prognosis and higher CD8+ T cells infiltration, as well as an activated antitumor immune response, while patients in IMP cluster_3 suffered from extremely poor prognosis, had higher infiltrations of potential immunosuppressive cells such as γδT cells, higher infiltrations of naive CD4^+^ T cells, and lower infiltrations of activated NK cells. The distribution of certain immune cells among IMPs has controversial results with previous studies. We considered that this may be due to the impact of the IMP clustering, or because of the potential limitation of bulk deconvolution estimation. The pan‐cancer TME functional signature analysis revealed significant concordant relationships among the three IMP clusters, IMP_Risk level, and four TME patterns in OS patients. IMP cluster_2 represented 67% and 56% of TME_IE and IE/F pattern with better prognosis, respectively, which were more enriched in the low‐IMP_Risk score group; while the high‐IMP_Risk group contained most of the IMP cluster_3 and higher proportion of TME_D pattern with poor prognosis.

Through the TIDE algorithm and T‐cell inflamed signature (TIS) analyses, we also evaluated the predictability of the IMP_Risk score in response to immunotherapy. Although there was no significant difference of overall TIDE scores between high‐ and low‐IMP_Risk groups, higher T‐cell exclusion scores, lower expression of the IFNG signature, and higher correlation with M2 TAM, MDSCs, and CAFs signatures, along with lower TIS scores, were found in the high‐risk group. Thus, the IMP_Risk score was compatible with a TME pattern to determine the immune cells infiltrations and functions, suggesting that the poor prognosis of the high‐risk group may result from the stronger immunosuppressive TME, and thus high‐risk patients may not benefit from ICI therapy. Nonetheless, even though our study should show new insights into the epigenomic microenvironment and possible IMP‐related therapies, many of our findings were based on retrospective studies. It is important to carry out prospective studies to validate and/or optimize the IMP‐associated signature scoring model. Ultimately, detailed functional and mechanistic studies of the signature genes in our risk model are highly warranted in order to support their clinical diagnostic and prognostic applications.

## 5 Conclusions

Our integrated analysis of immune‐related DNA methylomic and transcriptomic profiles revealed an extensive regulatory interconnection underlying the effects of TME and its relationship with OS prognosis. We constructed and validated an IMP‐associated signature scoring model, documented the crosstalk and regulatory roles of the signature genes in transcription and somatic copy number alteration, and identified their potential utilities in targeted therapy and immunotherapy. To the best of our knowledge, our study represents the first of its kind by focusing on a prognostic model incorporating IMP and TME patterns in OS. Our work also highlights the crucial clinical implications of DNA methylation in the crosstalk between tumor cells and TME, and should aid our efforts on developing personalized immune therapeutic strategies for OS patients.

## Conflict of interest

The authors declare no conflicts of interest.

## Author contributions

DS, ZZ, ZS, and T‐CH conceived and jointly supervised the study. DS, SM, FP, and ZZ collected data and conducted statistical analysis. JL, BZ, BH, NN, and HW contributed to statistical analysis and figure generation for the article. DS, SM, ZZ, FP, ZS, T‐CH, RCH, HHL, and LS drafted and revised the article. All authors reviewed and approved the article.

## Supporting information


**Fig. S1.** Supplementary plots for the analysis of immune‐related DNA methylation patterns in osteosarcoma. (a & b). Process of the unsupervised consensus clustering of Methylation beta value matrix of the immune‐related CpG sites in TARGET OS cohort and silhouette analysis. (c). Age, gender and race distributions of OS patients among three IMPs. (d, e & f). Validation of the IMP in E‐MTAB‐9875 cohort and silhouette analysis. (g, h & i). Validation of the IMP in E‐MTAB‐7263 cohort and silhouette analysis. (j). Relative proportion of immune infiltrating cells patients with OS of three IMPs and infiltrating scores of each type of immune infiltrating cells analyzed by quanTIseq. (k). Overall tumor microenvironmental infiltration scores (including stromal and immune) of patients with OS of three IMPs analyzed by xCell. (l). Kaplan‐Meier survival analysis of TME cells infiltration levels.

**Fig. S2.** Supplementary plots for construction and validation of an IMP‐associated signature scoring model in osteosarcoma. (a). A heatmap of methylation levels (z‐score) of differentially methylated probes between cluster 3 and cluster 2 of IMP. (b). A volcano plot differentially expressed genes between cluster 3 and cluster 2 of IMP. (c). a plot of median‐survival time of the TARGET OS cohort. (d). Kaplan‐Meier survival analysis using median IMP_Risk scores as the cutoff value displayed high‐risk OS patients with shorter overall survival. (e). distribution of IMP Risk scores and patients' survival status between IMP Risk subgroups. (f). time‐dependent ROC curve and Kaplan‐Meier analyses of the IMP‐associated signature scoring model (IMP Risk scores) on relapse‐free survival for TARGET OS cohort. (g). a plot of median‐survival time of the GSE21257 osteosarcoma cohort. (h & i). Validation of IMP‐associated signature scoring model on overall survival and metastasis‐free survival of GSE21257 osteosarcoma cohort through time‐dependent ROC curve and Kaplan‐Meier analyses. (j). Evaluation of the nomogram by calibration plots. (k). Decision curve analysis for the evaluation of prognostic predictors, including histologic response to chemotherapy, metastasis state, IMP‐associated signature scoring model.

**Fig. S3.** Supplementary plots for integrative analysis of molecular characteristics of osteosarcoma and IMP‐associated signature scoring model. (a). correlation analysis of gene expression level and DNA methylation level at the promoter region of the genes in IMP‐associated signature scoring model (supplementary information of *MT1A* and *MYC*). (b). A volcano plot differentially expressed genes between IMP Risk subgroups. (c). GSEA of altered hallmark, GO biological process, KEGG and Reactome pathway gene sets between IMP Risk subgroups.

**Fig. S4.** Supplementary plots for analysis of TME patterns in osteosarcoma and predictive value of the IMP‐associated signature scoring model on immunotherapy. (a). Process of the unsupervised consensus clustering of the robustly standardized GSVA enrichment scores of TME‐pattern signature gene sets. (b). The tumor immune dysfunction and exclusion (TIDE) scores of high‐ and low‐IMP Risk subgroups evaluated by TIDE algorithm. (c). Levels of cytotoxic tumor lymphocytes of high‐ and low‐IMP Risk subgroups evaluated by TIDE algorithm. (d). a plot of median‐survival time of the IMvigor210 cohort.

**Table S1.** Clinical information of patients included in the present study and the Immunologic signature. (Sheet 1‐4). Clinical information of patients included in the present study from TARGET osteosarcoma dataset, GSE21257, E‐MTAB‐9875, and E‐MTAB‐7263 cohorts. (Sheet 5). Immunologic signature gene sets.

**Table S2.** Univariate cox regression analysis of overall survival based on CpG probes and the result of consensus clustering. (Sheet 1). Univariate cox regression analysis on methylation levels (Beta value) of CpG sites. (Sheet 2). Annotation of immunologic signature‐related CpG sites. (Sheet 3). Result of consensus clustering.

**Table S3.** Estimation of TME‐infiltrating cells and the GSVA results. (Sheet 1‐3.) Quantitative results of CIBERSORTx, quanTIseq and xCell on TME‐infiltrating cells and overall TME/stromal/immune infiltration scores. (Sheet 4‐6.) Enrichment scores of GSVA of each patient of the three IMPs.

**Table S4.** Analysis of DMP, DEG, and cis‐/trans‐ regulation. (Sheet 1‐2.) Results of DEG and DMP analyses (IMP cluster_3 vs cluster_2). (Sheet 3‐4.) Pearson analyses on cis‐ and trans‐ regulation for gene expression and DNA methylation of CpG sites.

**Table S5.** Statistical results for the construction and validation of the IMP‐associated signature scoring model.

**Table S6.** Analysis of molecular characteristics and pharmacogenomic data associated with IMP‐associated signature scores. (Sheet 1‐2.) CpG sites relevant to the six genes that comprise the IMP‐associated signature scoring model (cis‐ or trans‐ regulation). (Sheet 3‐8.) Results of DEG and preranked GSEA analyses of high‐ and low‐IMP_Risk groups. (Sheet 9‐13.) Identification of IMP_Risk score highly associating genes through Pearson correlation analysis. Results of over‐representation analysis and PPI network analysis.(Sheet 14‐17.) Results of GISTIC 2.0 on SCNA of high‐ and low‐IMP_Risk groups.(Sheet 18.) Spearman correlation analysis on drug response (AUC values) and IMP scores based on GDSC datasets.

**Table S7.** Analysis of TME pattern and prediction of immunotherapy response. (Sheet 1‐3.) GSVA of the pan‐cancer TME functional signatures and consensus clustering of four TME patterns. (Sheet 4‐6.) Gene symbols of T‐cell inflamed signature (TIS), results of TIDE analysis, and information of the IMvigor210 cohort including the IMP_Risk scores.

## Data Availability

The RNA sequencing or microarray data, clinical information and gene sets analyzed in this study are described in the methods section “Data selection and acquisition.” The resources, tools, and r packages used in our study are described in each section in the methods. Processed data are included in supplementary files.
