# Supplementary figures and images for: Integrative analysis of immune‐related multi‐omics profiles identifies distinct prognosis and tumor microenvironment patterns in osteosarcoma
Source: Mol Oncol. 2022 Jan 1;16(11):2174–94. doi: 10.1002/1878-0261.13160 (PMC9168968; doi:10.1002/1878-0261.13160)

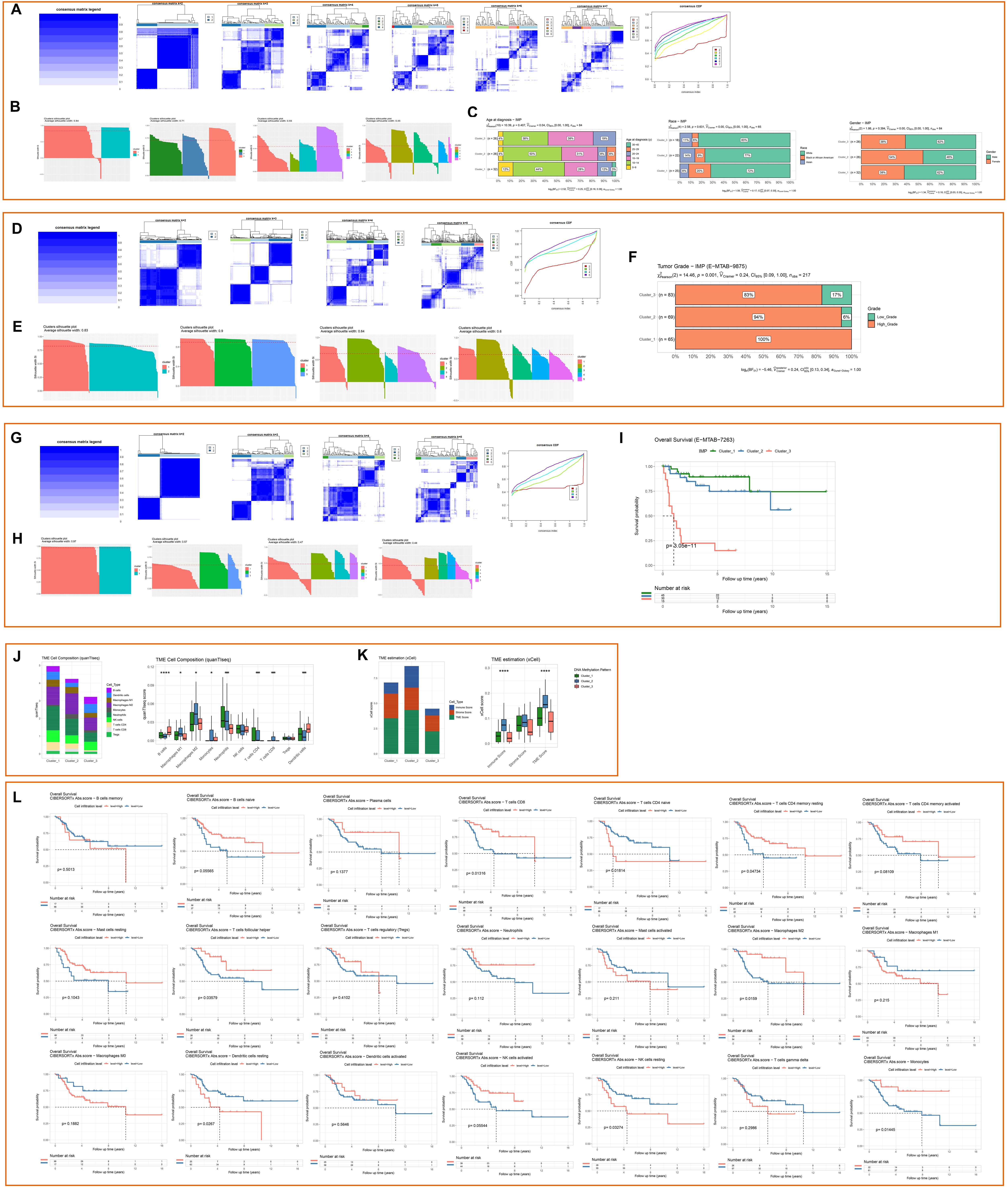

Supplement: Supplementary file 1 — Fig. S1. Supplementary plots for the analysis of immune‐related DNA methylation patterns in osteosarcoma. (a & b). Process of the unsupervised consensus clustering of Methylation beta value matrix of the immune‐related CpG sites in TARGET OS cohort and silhouette analysis. (c). Age, gender and race distributions of OS patients among three IMPs. (d, e & f). Validation of the IMP in E‐MTAB‐9875 cohort and silhouette analysis. (g, h & i). Validation of the IMP in E‐MTAB‐7263 cohort and silhouette analysis. (j). Relative proportion of immune infiltrating cells patients with OS of three IMPs and infiltrating scores of each type of immune infiltrating cells analyzed by quanTIseq. (k). Overall tumor microenvironmental infiltration scores (including stromal and immune) of patients with OS of three IMPs analyzed by xCell. (l). Kaplan‐Meier survival analysis of TME cells infiltration levels. [file MOL2-16-2174-s007.tif]

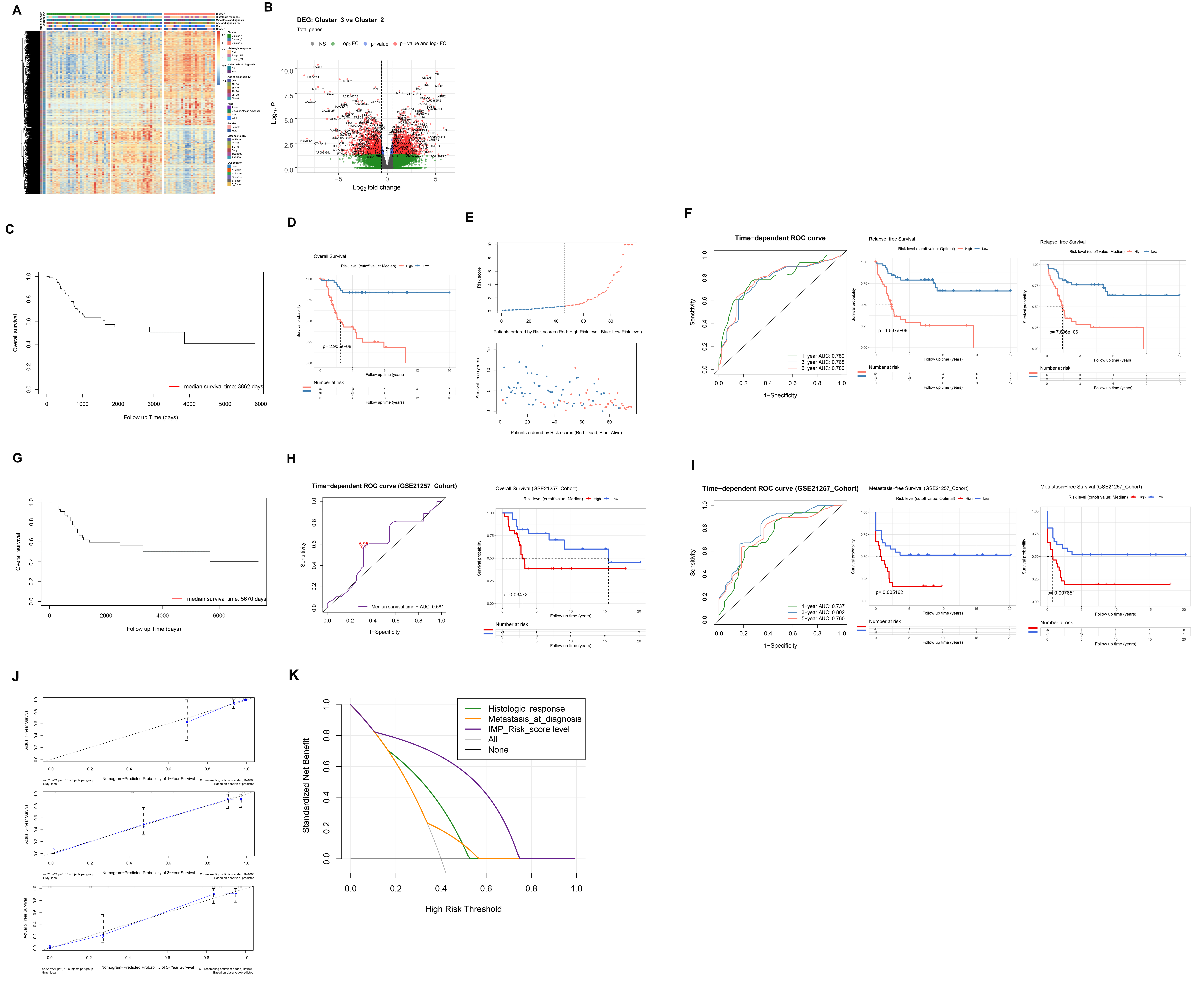

Supplement: Supplementary file 2 — Fig. S2. Supplementary plots for construction and validation of an IMP‐associated signature scoring model in osteosarcoma. (a). A heatmap of methylation levels (z‐score) of differentially methylated probes between cluster 3 and cluster 2 of IMP. (b). A volcano plot differentially expressed genes between cluster 3 and cluster 2 of IMP. (c). a plot of median‐survival time of the TARGET OS cohort. (d). Kaplan‐Meier survival analysis using median IMP_Risk scores as the cutoff value displayed high‐risk OS patients with shorter overall survival. (e). distribution of IMP Risk scores and patients' survival status between IMP Risk subgroups. (f). time‐dependent ROC curve and Kaplan‐Meier analyses of the IMP‐associated signature scoring model (IMP Risk scores) on relapse‐free survival for TARGET OS cohort. (g). a plot of median‐survival time of the GSE21257 osteosarcoma cohort. (h & i). Validation of IMP‐associated signature scoring model on overall survival and metastasis‐free survival of GSE21257 osteosarcoma cohort through time‐dependent ROC curve and Kaplan‐Meier analyses. (j). Evaluation of the nomogram by calibration plots. (k). Decision curve analysis for the evaluation of prognostic predictors, including histologic response to chemotherapy, metastasis state, IMP‐associated signature scoring model. [file MOL2-16-2174-s010.tif]

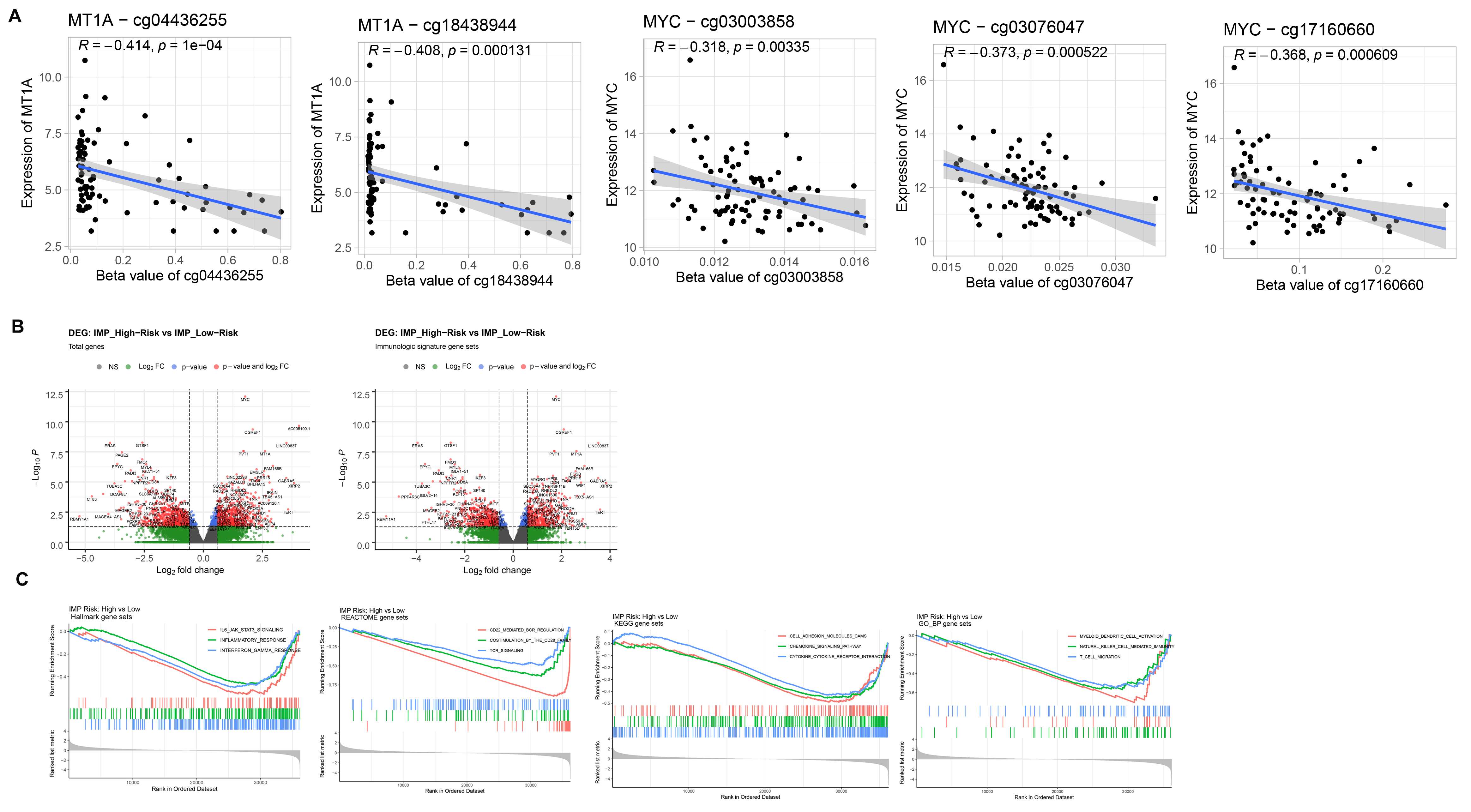

Supplement: Supplementary file 3 — Fig. S3. Supplementary plots for integrative analysis of molecular characteristics of osteosarcoma and IMP‐associated signature scoring model. (a). correlation analysis of gene expression level and DNA methylation level at the promoter region of the genes in IMP‐associated signature scoring model (supplementary information of MT1A and MYC). (b). A volcano plot differentially expressed genes between IMP Risk subgroups. (c). GSEA of altered hallmark, GO biological process, KEGG and Reactome pathway gene sets between IMP Risk subgroups. [file MOL2-16-2174-s008.tif]

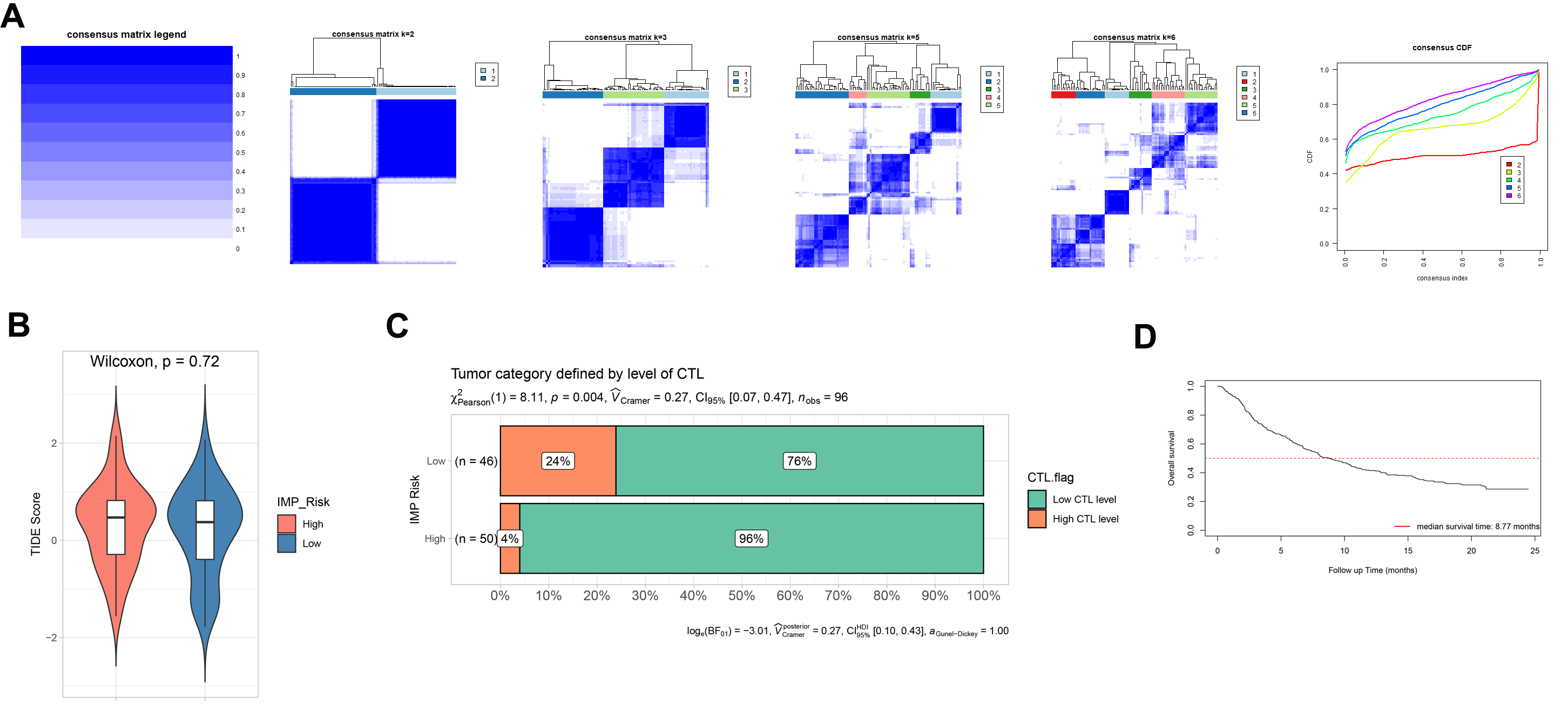

Supplement: Supplementary file 4 — Fig. S4. Supplementary plots for analysis of TME patterns in osteosarcoma and predictive value of the IMP‐associated signature scoring model on immunotherapy. (a). Process of the unsupervised consensus clustering of the robustly standardized GSVA enrichment scores of TME‐pattern signature gene sets. (b). The tumor immune dysfunction and exclusion (TIDE) scores of high‐ and low‐IMP Risk subgroups evaluated by TIDE algorithm. (c). Levels of cytotoxic tumor lymphocytes of high‐ and low‐IMP Risk subgroups evaluated by TIDE algorithm. (d). a plot of median‐survival time of the IMvigor210 cohort. [file MOL2-16-2174-s002.tif]
